# Pontine Infarct Resulting in Millard-Gubler Syndrome: A Case Report

**DOI:** 10.7759/cureus.34869

**Published:** 2023-02-11

**Authors:** Shivangi Patel, Ashika Bhakta, Joshua Wortsman, Barun B Aryal, Dhan B Shrestha, Tilak Joshi

**Affiliations:** 1 Internal Medicine, Ross University School of Medicine Barbados Campus, Barbados, BRB; 2 Department of Internal Medicine, Mount Sinai Hospital, Chicago, USA; 3 Department of Medicine, Mount Sinai Hospital, Chicago, USA

**Keywords:** pontine infarct, case report, facial nerve palsy, hemiparesis, millard-gubler syndrome

## Abstract

Millard-Gubler syndrome is a crossed brainstem syndrome involving the facial nerve, abducens nerve, and the pyramidal tracts, typically resulting in ipsilateral facial weakness and contralateral hemiparesis. Here we report the case of a 76-year-old female with no pertinent past medical history who presented to the emergency department with acute left-sided facial droop and right upper extremity sensory loss. A pontine infarction was identified on imaging and she was managed medically with complete recovery. Pontine infarction can result in Millard-Gubler syndrome and present with facial weakness and subtle contralateral limb symptoms.

## Introduction

Millard-Gubler syndrome is a rare ventral pontine syndrome that manifests with ipsilateral weakness of the eye upon abduction (abducens nerve), ipsilateral facial muscle weakness (facial nerve), and contralateral hemiparesis or hemiplegia of the upper and lower extremities (pyramidal tract fibers) [1-3]. This is usually due to unilateral lesion at the basal portion of the caudal pons as a result of a mass, hemorrhage, or rarely, infarction [4-7]. Hemorrhage and infarction are more common in older patients, while tumors and infections are more common in younger individuals.

Bell's palsy is a result of an acute idiopathic peripheral facial nerve palsy due to a unilateral lower motor neuron lesion between the facial nerve nuclei and muscles. In this case report, we present a patient with acute onset facial droop with no apparent cause. Extensive workup was done to determine the presence of stroke and potential arterial stenosis. The patient was found to have an acute infarction at the left dorsal brainstem at the pontomedullary junction which, along with her clinical presentation, pointed to the diagnosis of Millard-Gubler syndrome.

## Case presentation

A 76-year-old female with no significant past medical history presented to the emergency department with a left-sided facial droop that began one day prior and had been worsening. She also complained of numbness in her right fingers, which had resolved by the time of the presentation. She denied weakness in upper and lower extremities, slurred speech, and changes in gait. She also denied fever, chills, headache, changes in vision, chest pain, palpitations, shortness of breath, abdominal pain, nausea, vomiting, or dysuria. She denied any history of recent trauma, travel, or recent upper respiratory tract infection. She had never experienced similar symptoms before. Her family history was non-contributory.

Upon arrival, she was afebrile, had a heart rate of 123, respiratory rate of 22, blood pressure of 191/103, and oxygen saturation of 96% on room air. On examination, her pupils were equal, round, and reactive to light and accommodation. Extraocular movements were intact bilaterally. Facial sensation was intact, but there was a left upper and lower facial palsy with decreased prominence of the left nasolabial fold. Motor and sensory examination of bilateral upper and lower extremities were normal and reflexes were intact. She was oriented to person, place, time, and situation and her speech was normal. The rest of the physical examination was unremarkable. Laboratory investigations showed no significant electrolyte abnormalities.

Due to concern for acute or subacute stroke, a non-contrast computed tomography angiography of the head and neck was done. This revealed a sub-centimeter hypodensity in the left lentiform nucleus, reflecting an age-indeterminate lacunar infarct. There was also mild stenosis of the proximal basilar artery and moderate stenosis of the origin of the right posterior cerebral artery. There were no signs of hemorrhage. She was given aspirin and atorvastatin in the emergency room. Permissive hypertension was maintained for 24 hours and blood pressure was then managed with oral antihypertensive agents. Thrombolysis was not indicated since she presented more than 4.5 hours after the onset of symptoms.

**Figure 1:**
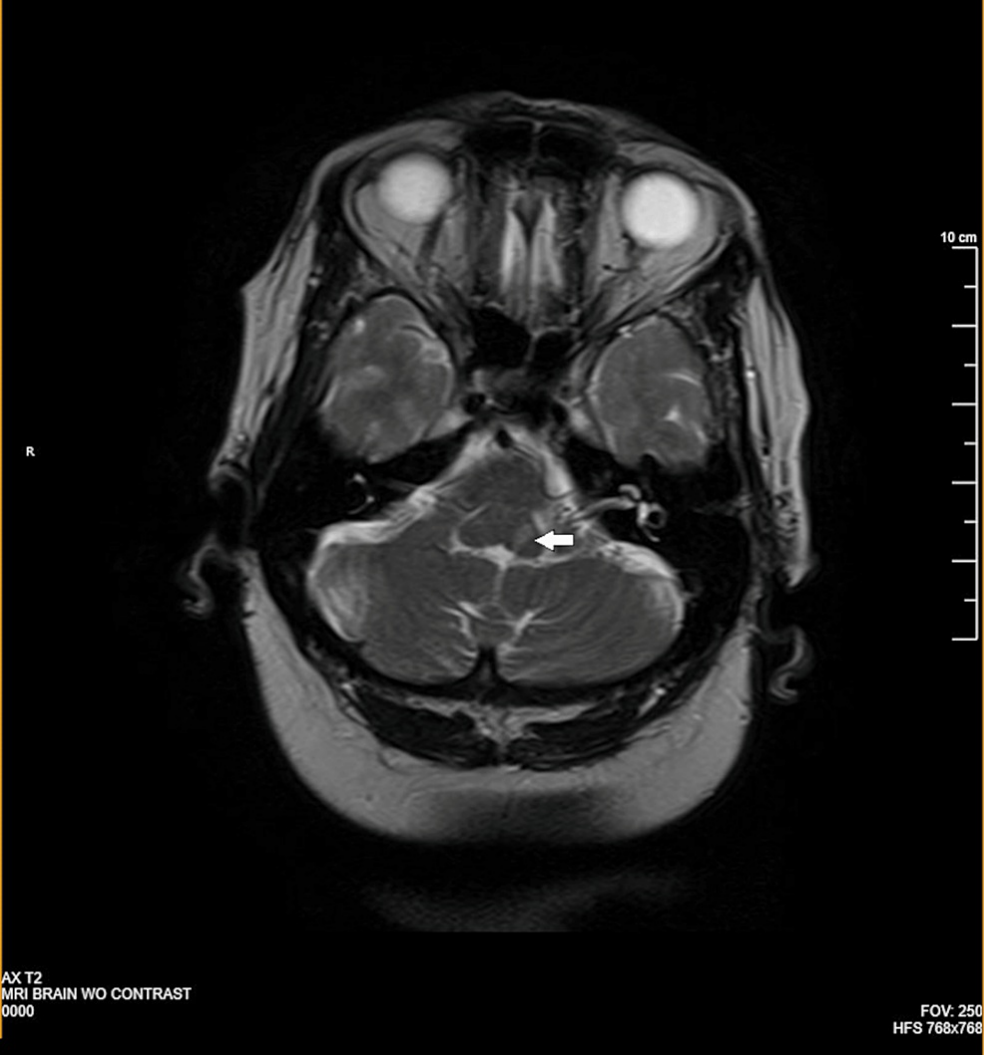
MRI brain showing small left dorsal brainstem infarct at the pontomedullary junction (white arrow)

For better visualization of the lesion, a magnetic resonance imaging of the brain was ordered, which revealed a small infarct in the left dorsal brainstem at the pontomedullary junction consistent with an acute infarction, as seen in Figure 1.

The patient was subsequently admitted for management of an ischemic stroke. Differential diagnoses included Bell’s palsy and Millard-Gubler syndrome. Serological tests for Hepatitis B, Hepatitis C and HIV were negative. In order to rule out Lyme disease as the cause of facial nerve palsy, Lyme disease IgG/IgM antibodies were ordered, which came back negative. The diagnosis of Millard-Gubler syndrome was made based on imaging findings of acute pontine infarction and neurological findings of ipsilateral facial palsy.

She was treated with dual antiplatelet therapy with aspirin and clopidogrel. Physical rehabilitation was initiated. By the eighth day of admission, her symptoms had completely resolved. She was then discharged, and after 21 days of dual anti-platelet therapy, she was switched to aspirin alone with the addition of a statin. On follow-up after eight weeks, she had no residual symptoms or new neurological symptoms.

## Discussion

We presented the case of a 76-year-old female who presented with new-onset facial droop and decreased sensation in the upper right extremity which had resolved by itself. On imaging, a small acute infarction was identified in the left pontomedullary junction. A diagnosis of Millard-Gubler syndrome was made, and she recovered fully with physical rehabilitation.

Millard-Gubler syndrome, also known as ventral pontine syndrome or facial abducens hemiplegia syndrome, was first described in 1858, and was classically associated with a pontine mass [8]. Subsequent reports, however, have involved cases caused by bleeding and infarcts, and rarely neurocysticercosis [2-7,9,10]. The lesion lies above the level of the decussation of the pyramidal and spinothalamic tracts. As a result, the cranial nerve signs are ipsilateral whereas the limb symptoms are contralateral, resulting in the classical crossed brain stem syndrome [3].

The involvement of the pyramidal tracts usually manifests in hemiplegia or hemiparesis of upper or lower extremities in addition to facial palsy [2-5,10]. In our case, there was no motor deficit in the extremities. The patient complained of right-sided upper extremity numbness which had resolved by the time of examination, and no sensory deficit in the extremities was present on initial or subsequent examinations.

Unilateral facial weakness was prominent in this case, and the presentation was typical of peripheral facial palsy, with involvement of upper and lower face. One of the alternative diagnoses considered was Bell's palsy, which is a diagnosis of exclusion. However, presence of contralateral sensory symptoms, albeit transient, would not be expected in Bell's palsy. Other intracranial pathology such as tumors, vascular malformations, and neurocysticercosis were excluded via imaging. Herpes zoster reactivation is another potential cause of facial nerve palsy but would present with pain and vesicles on examination in affected dermatomes, which were not present in this case. Moreover, evidence of acute pontine infarction on imaging confirmed the diagnosis of Millard-Gubler syndrome.

Our patient had a full recovery within one week of presentation. This is consistent with the clinical course described in case reports, especially those involving small acute infarcts [2,4,6,7,9,10].

Brainstem infarcts are usually seen in the background of various risk factors such as hypertension, diabetes, and hyperlipidemia. Our patient had previously undiagnosed hypertension but no other significant medical history. Our case demonstrates the utility of imaging in diagnosing Millard-Gubler syndrome and distinguishing it from other differential diagnoses, especially in cases with an unconventional patient profile and presentation.

## Conclusions

Millard-Gubler syndrome is a rare neurological syndrome resulting from pontine infarction. It can present with facial weakness and contralateral limb symptoms, which can sometimes be subtle or self-resolving. MR imaging can be very helpful in confirming the diagnosis and differentiating it from other differential diagnoses such as tumors, vascular malformations or other intracranial masses. Complete recovery is possible with supportive management.
